# Survey on Public Psychological Intervention Demand and Influence Factors Analysis

**DOI:** 10.3390/ijerph18094808

**Published:** 2021-04-30

**Authors:** Fang Su, Bingjie Fan, Nini Song, Xue Dong, Yanxia Wang, Jingzhong Li, Bing Xue, Xianrong Qiao

**Affiliations:** 1 School of Economics and Management, Shaanxi University of Science & Technology, Xi’an 710000, China; bingjie2122@163.com (B.F.); songnini1020@sina.com (N.S.); 2 Mental Health Education Center, School of Education, Shaanxi University of Science and Technology, Xi’an 710000, China; dongxue@sust.edu.cn; 3 Department of Scientific Research Center, Gansu Provincial Maternity and Child-Care Hospital, Lanzhou 730050, China; evawyx@163.com; 4 School of Urban Planning and Landscape Architecture, Xuchang University, Xuchang 461000, China; zhong_lij@163.com; 5 Key Lab of Pollution Ecology & Environmental Engineering, Institute of Applied Ecology, Chinese Academy of Sciences, Shenyang 110016, China; xuebing@iae.ac.cn; 6 Arts and Sciences School, Translation and Cultural Communication Research Institute, Shaanxi University of Science and Technology, Xi’an 710000, China; qiaoxianrong@sust.edu.cn

**Keywords:** emergency management, mental health, crisis management, psychological intervention

## Abstract

Major public health emergencies would have a negative influence on the psychology of the public, and an effective psychological intervention can help them to relieve some emotions, such as tension and panic. However, differences in individual environments affect people’s psychological intervention demands and intervention mode choices. Therefore, it is of great theoretical and practical value to analyze and identify the key factors affecting these demands and choices. Based on a nationwide sample of 24,188 respondents from the “Internet Survey of Residents’ Behavioral Changes and Psychological Conditions during the Epidemic”, the different characteristics of public psychological intervention demands and choices under different factors are explored in this paper. The results demonstrate that: (1) the psychological status of Chinese people was relatively stable during the epidemic period, and there were 1016 respondents who had subjective demands for a psychological intervention, (2) age, gender, occupation type, residence, family size, risk perception, psychological status, education level, and fixed expenditure all significantly affect public psychological intervention demands, and (3) risk perception, psychological status, age, gender, and family size will impact the choice of psychological intervention methods. The above results can provide a decision-making basis for the construction of a psychological intervention system in psychological crisis management during the post-epidemic prevention and control period, as well as reference and suggestions for handling psychological stress of similar sudden crisis events in the future.

## 1. Introduction

In January 2020, the World Health Organization (WHO) declared the Corona Virus Disease-19 (COVID-19) caused by SARS-CoV-2 as a public health emergency of international concern [1]. The occurrence of such major stress events will have a profound impact on all areas of social life [2]. Meanwhile, it will also pose a huge influence on public psychology, resulting in a series of mental and psychological problems (such as the change of personal emotion, cognition, and behavioral activities) and, thus, affect social stability [3].

Xi Jinping, President of China, pointed out that the COVID-19 outbreak is a major public health emergency with the widest influenced area and the fastest spreading speed happened since the founding of the People’s Republic of China, its prevention and control are the most difficult [4], and this clearly indicates that “the government should strengthen psychological counseling and psychological intervention, enhance public opinion guidance, and properly handle the various problems that may occur in the epidemic prevention and control, so as to safeguard overall social stability” [5]. The psychological state of the public in the emergency period is often the concentrated reflection of the maturity and civilization of the public in the society [6]. Meanwhile, a perfect modern emergency management system should also focus on the social psychological behavior of the public [7]. Therefore, psychological intervention management for major public events has become a major issue for the governance capacity of the government and the construction of a shared human future. Innovation on the study of the psychological intervention based on the diversity of the public demand and psychological intervention mode choice has an important theoretical value and practical significance for us to explore an effective psychological intervention strategy and improve the national emergency management system.

Based on the previously mentioned reasons and the COVID-19 information stimulation background, this study carried out a questionnaire survey on the epidemic-related behavioral characteristics, psychological stress level, psychological intervention demands, and mode choices, aiming to explore people’s psychological features and the law of the psychological intervention demand and mode selection during the COVID-19 outbreak. It is to provide a reference basis for relevant disease prevention and control departments and medical institutions to formulate psychological intervention strategies for public health emergencies, and also provide some scientific support and a decision-making reference for the construction of a long-term psychological intervention mechanism with differentiated social attributes.

## 2. Literature Review

Grave emergencies, as a stress source, including fires, floods, earthquakes, and outbreaks of infectious diseases, are highly unexpected, uncertain, and widespread. Once it occurs in densely populated areas, it will not only cause damage to the public’s physical health but also inflict some long-lasting harm on their psychological health [8]. Present studies have demonstrated that the mental state of the public is more likely to change abruptly and suffers severe psychological stress when a public health emergency occurs in an area and the epidemic situation is gradually emerging. In this case, its main manifestations are anxiety and fear, feeling unwell, having cognitive difficulties, and losing a sense of security with compulsive characteristics and fragile interpersonal relationships [9,10,11,12,13,14]. Facing critical emergencies, the public will have a stress reaction that will directly or indirectly damage their cognition, emotion, and behavior. Specifically, the higher the level of anxiety, the stronger the psychological pressure reaction [15].

As the victims and responders of the crisis, it is crucial to solve the psychological crisis and promote social stability to help the public alleviate the psychological stress injury and the adverse psychological mood [16]. Studies have indicated that psychological intervention plays an imperative role in the process of handling major emergencies, and has significant effects on reducing somatization symptoms, obsessive symptoms, anxiety, and depression, relieving interpersonal sensitivity, and improving hostile emotions [17]. Effective psychological intervention can help the public regain a sense of physical and psychological security and restore a state of psychological balance [18,19]. However, there are certain differences in the stress response for different individuals under the same stressor (molecular biochemical reaction, individual hormonal changes, and external behavior, emotion, and cognitive changes) [20]. Individuals of different ages, genders, educational levels, and incomes present great differences in their living environment and psychology, and their psychological intervention demands would be affected by different factors [21]. Therefore, targeted and differentiated psychological intervention measures for different individuals should be adopted to achieve better effects of psychological intervention and improve the effectiveness of psychological intervention [22].

At present, psychological research on major epidemic events in China has recently started, and there are few localized research results. The existing research mainly focuses on the changes in the psychological state of the public, the representation of psychological stress, and the choice of psychological intervention methods under public health emergencies. The commonly used social psychological intervention methods primarily include an intervention against the cognitive bias of patients, building a good nursery-patient relationship, opening a psychological counseling hotline, providing emotional support group counseling, and establishing a social support system [23,24,25,26,27]. Overall, the current methods of psychological intervention in China are based on emotions and characterized by mental representation of patients, group intervention, and passive intervention, less attention to the public demand for psychological intervention or subjective demands for psychological intervention, and a lack of special treatment for different groups after major emergency psychological intervention mode selection under individual differences. Thus, there is a constraint on the strategy.

In this study, based on the psychological stress status of Chinese people and their subjective demands for psychological intervention during the COVID-19 epidemic, a study combining people’s subjective will for psychological intervention and choice of intervention methods was conducted to explore the influence of factors, such as demographic characteristics, a psychological status, and risk perception on public psychological intervention demands and choice of intervention methods. The results provide scientific guidance and a decision-making basis for the construction of a long-term mechanism of individual psychological intervention.

## 3. Data Resources and Methodology

### 3.1. Data Collection and Participants 

From 21 February to 28 February 2020, the research team conducted *An Investigation on Residents’ Living Behavior and Psychological State during the Pandemic* based on the individual behavior characteristics, psychological stress status, and psychological intervention demand of the Chinese public. The data in this paper mainly originate from this social investigation. The “*Questionnaire Star*” network platform and the non-probabilistic snowball subjective sampling method were adopted in this survey according to the principle of comprehensibility, timeliness, and convenience. First, each member of the research team sought 30 respondents who met the requirements to fill in the electronic questionnaire. Then, these respondents forwarded the questionnaire link to other objects who met the requirements around them. Afterward, the system automatically saved the record. All respondents voluntarily filled in the form and signed the informed consent.

The reliability and validity of the questionnaire were tested by three psychological experts and two sociological experts, who met the requirements of statistics, to ensure the quality and science of the questionnaire. Simultaneously, the following measures were adopted in the investigation. (1) Before the formal survey, team members tried to fill in the questionnaire many times and timely corrected errors. (2) The same IP and device of the electronic questionnaire can only be filled in once. (3) Common sense questions irrelevant to the research were set in the questionnaire, and the wrong answers to common sense questions were eliminated. (4) The filled questionnaire will be eliminated if filled within 8 min (the normal filling time of the questionnaire is 8–10 min). Finally, a total of 24,215 samples were collected, of which 24,188 were valid, with an effective rate of 99.89%.

### 3.2. Research Design

#### 3.2.1. Evaluation of the Psychological States

Psychologist Levitov believes that the individual mental state is affected by factors such as environment, psychological activities, emotions, and typical events, which are both temporary and stable [28]. Based on existing studies [28,29], the temporary psychological stress of Chinese people in the context of COVID-19, was investigated in this paper. Specifically, the questionnaire mainly involved psychological anxiety, psychological depression, and psychological stress, as follows.

***Questionnaire Setting of Psychological Status Assessment.***

*Question 1: Do you feel anxious/depressed about the grim situation of the epidemic?*
*A. YES*                    
*B. NO*



*Question 2: Are you frustrated/upset because you cannot work or live a normal life during the outbreak?*
*A. YES*                    
*B. NO*



*Question 3: If the maximum value of stress is a score of 100, which of the following ranges would you self-rate as psychological stress in the current environment?*
*A. 1–30*     *B. 30–50*     *C. 50–80*     
*D. 80–100*



The quantification method is:

For Questions 1 and 2, If the respondents choose “yes,” they will be scored 1 point. If the respondents chooses “No,” the score is 0. For Question 3, if the self-assessed psychological stress value of the interviewees ranges from “1–30,” it is scored as 0 points. If it is in the range of “30–50,” it is scored 1 point. If it is in the range of “50–80,” it is scored 2 points. if it is in the range of “80–100,” it is scored 3 points.

The comprehensive evaluation method was employed to evaluate the psychological status of the public during the epidemic [30], and the final score was between 1 and 5 points. The higher the score, the greater the psychological pressure of the public.

#### 3.2.2. Model Setting for Psychological Intervention Demands

Schachter (1964) believed that emotions were generated by integrating stimulus factors, physiological factors, and cognitive factors [31]. Based on this, a binomial Logistic regression analysis model was built and the study reveals the influence of the individual characteristics, psychological status, and risk awareness on public psychological intervention demand. The main influential variables include gender, age, level of education, residential area, type of occupation, family size, risk perception, psychological status, and accumulative number of confirmed patients. The specific setting is:

(1) $$\log it\left(p\right)=\ln\left(\frac{p}{1-p}\right)=\beta_{0}+\beta_{1}x_{1}+\beta_{2}x_{2}+\cdots \cdots \beta_{12}x_{12}+\mu$$

(2) $$odds=\frac{p}{1-p}=\exp\left(\beta_{0}+\beta_{1}x_{1}+\beta_{2}x_{2}+\cdots \cdots +\beta_{12}x_{12}\right)+\mu$$

where *p* refers to the psychological intervention demand of the respondent, namely the probability of “need = 1” occurs. $\beta_{0}$ represents the regression constant. $\beta_{j}$ (j = 1, 2, …, 12) denotes the regression coefficient. μ is the random external excitation. $x_{1}$, $x_{2}$, …, $x_{12}$ indicate the variables affecting people’s demand for psychological intervention. The setting and explanation of the various variates in the specific model are provided in Table 1.

#### 3.2.3. Model Setting for Selection of Psychological Intervention Methods

Based on existing research and the actual situation of current epidemic prevention and control in China [23,32,33], this research provides respondents with six selections.

(1) Self-persuasion, referring to individual engagement in psychological counseling activities including reading, meditation, and exercise diverting attention and calm the mood. (2) Psychological counseling under professional guidance, referring to how individuals release their inner pressure under the guidance of professionals through one-on-one communication. (3) Communication and counseling among homogeneous groups, in which the groups may include classmates and colleagues similar in certain aspects such as age, experience, intelligence, and ability. They may communicate with each other. (4) “Individual-social” family relationship communication facilitation, referring to individuals’ communicating and counseling with family members and relatives. (5) Other types of counseling, referring to the method used to relieve psychological pressure through religious faith and attending dharma assembly in this survey. (6) Do not understand the way of psychological counseling for the time being. This suggests that people do not know how to relieve psychological pressure or psychological tension at present.

It is assumed in this research that the psychological intervention methods needed are relatively independent. The single choice psychological intervention method is adopted as a dependent variable to further analyze the influencing factors of people’s subjective demands for the psychological intervention during the epidemic. The multivariate logistic regression analysis model is constructed to reveal the influence degree of different influencing factors on the psychological intervention selection.

(3) $$Ln\left[\frac{p\left(y=j/x\right)}{p\left(y=J/x\right)}\right]=\alpha_{j}+\sum_{K=1}^{K}\beta_{jk}x_{k}$$

where y denotes different psychological interventions. $x_{k}$ refers to the kth independent variable, including gender, age, education, fixed charge, family-scale, risk perception, and psychological states that can influence psychological intervention selection of the republic. $\beta_{jk}$ represents the variate of the independent variable regression coefficient. J indicates the reference type. The main variates are described in Table 2.

## 4. Results

### 4.1. Data Description

The basic information, such as their gender, age, education level, place of residence, type of occupation, family size, and whether they have a fixed income/fixed expenditure, are investigated to reveal the individual characteristics of the respondents. The descriptive statistics are listed in Table 3.

### 4.2. Psychological States

In this survey, the average psychological status of individuals was 3.266 points, and the mode was 4 points. The overall psychological status value exhibited a negative skew distribution, reflecting that the psychological pressure of the people during the epidemic period was greater. In total, 34.3% of the respondents felt anxious. In addition, 42.0% of them felt depressed or upset because they could not live and work normally during the epidemic period. Furthermore, 50.2% of them had more than 50 points of psychological stress and 12,143 of them were under high psychological pressure.

### 4.3. A General Picture of the Demands for a Psychological Intervention

**Figure 1:**
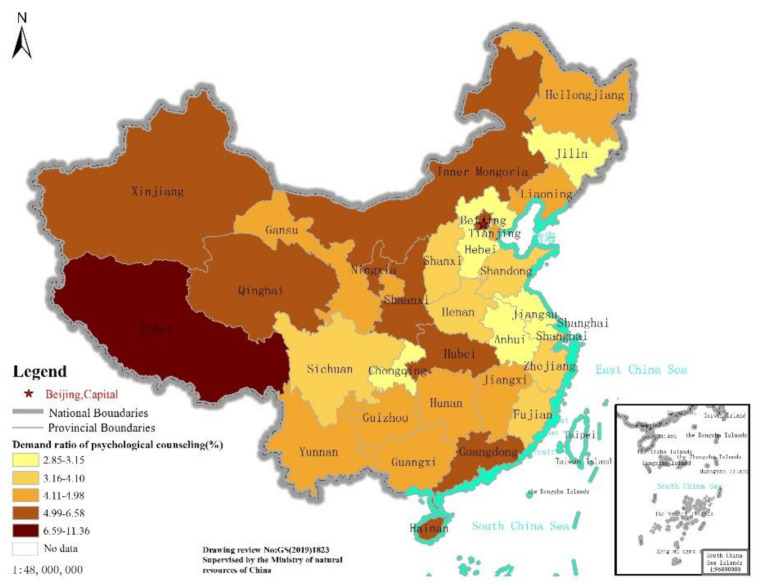
Distribution of people’s psychological counseling demands in China.

According to data analysis, of the 24,188 national samples, 1016 respondents needed a psychological intervention. As indicated by the regional distribution of psychological intervention demand, the total number of samples in the eastern region is 7095, and there are 314 individuals in need of intervention. The total number of samples in the central region is 5323, and there are 219 individuals in need of intervention. The total number of samples in the western region is 9407, and there are 433 individuals in need of intervention. The proportions of people who need psychological intervention are 4.42%, 4.11%, and 4.60%, respectively. In the provincial distribution of psychological intervention demand (Figure 1), Hubei Province, where the epidemic situation is concentrated, is not the region with the highest demand for a psychological intervention. Meanwhile, Tibet is the region with the highest demand for a psychological intervention.

A total of 1016 individuals with psychological demands were selected for psychological intervention (choose one or more). As is presented in Table 4, the proportions of individuals choosing self-persuasion and professional guidance are relatively high. For the majority of individuals, self-persuasion is still the preferred method of psychological intervention.

### 4.4. Influencing Factors of the Demands for Psychological Intervention

Based on 24,188 national samples, the influencing factors of whether people need psychological intervention were analyzed in this paper. According to the test of fitting degree of the model, the Chi-square value of the model is 13.173, and the *p*-value of the Hosmer-Leme test is over 0.05, indicating that the overall fitting effect of the model is acceptable, and the model results are reliable (Table 5).

The regression results and factors, such as age, gender, education level, occupation type, residence, family size, fixed charge, psychological status, and risk perception, can affect psychological intervention demands of the public.

“Psychological status” and “risk perception” are the significant factors affecting the “psychological intervention demand” of the public (*p* < 0.01). Regarding individual characteristics, “gender,” “education level,” and “fixed charge” have significant effects on “psychological intervention demand” (*p* < 0.01). The significant effects of “age,” “residence,” “occupation type,” and “family size” on “psychological intervention demands” are slightly weaker than the former (*p* < 0.1).

### 4.5. Influencing Factors of Psychological Intervention Methods

Based on 1016 data samples with psychological intervention demands, 552 surveyed samples with one certain psychological intervention method were selected for support. Meanwhile, with “do not understand psychological counseling methods or services” as a reference option, the influence factors and intensity of the psychological intervention methods of the public are revealed. In the model fitting information, *p* < 0.05 indicates that the model results are reliable (Table 6).

It can be observed from the regression results that:

The “psychological status” is a significant factor in the three types of counseling methods: “self-persuasion,” “psychological counseling under professional guidance,” and “communication and counseling among homogeneous groups” (*p* < 0.05). The “risk perception” is a significant factor affecting the public’s choice of “self-persuasion” (*p* < 0.01) and “communication and counseling among homogeneous groups” (*p* < 0.05).

“Age” has a significant negative effect on the choice of “communication and counseling among homogeneous groups” (*p* < 0.01). “Household scale” has a significant negative impact on the public’s choice of “other types of counseling (such as religion and dharma association)” (*p* < 0.05). “Gender” is a significant factor affecting “other types of counseling (such as religion and dharma association)” (*p* < 0.05).

## 5. Discussion

### 5.1. Psychological State and Distribution of Demands

From the perspective of the public’s psychological state assessment and the choice of psychological intervention methods, most individuals in China have a good overall psychological state while a small part of individuals has psychological intervention demands.

In terms of regional distribution, the western region (including Inner Mongolia, Guangxi, Chongqing, Sichuan, Guizhou, Yunnan, Shaanxi, Gansu, Qinghai, Ningxia, Xinjiang, and Tibet) presents a higher proportion of intervention demands than the other two regions. The main reasons are: (1) the western region is underdeveloped, (2) the medical and educational resources are relatively scarce, and (3) the changes in economy, life, and cognition stimulated by the epidemic situation are more likely to cause psychological pressure. However, in the eastern regions (including Beijing, Tianjin, Hebei, Liaoning, Shanghai, Jiangsu, Zhejiang, Fujian, Shandong, Guangdong, and Hainan) with sound medical and health systems and abundant educational resources, the possibility of epidemic spreading is increased due to the large population density and frequent population flow. Previous studies have shown that population density and a lack of medical equipment are key factors in the morbidity and mortality of COVID-19 [34]. In the present study, resource scarcity and population density are also critical elements influencing residents’ demands for psychological intervention, and resource-poor areas have greater needs for a psychological intervention.

Generally, the epidemic area should be the most concentrated area of psychological problems, and the level of its psychological intervention demands should be higher. The reason may be that, after the outbreak of the epidemic, the local government of Hubei province attached great importance to the psychological condition of the public in the epidemic concentrated areas, and carried out relevant psychological counseling and intervention work in time [35]. To some extent, these measures appeased the psychological stress reaction of the public and reduced their psychological intervention demands. However, Tibet is located in Western China, where the medical and educational resources are relatively scarce. The severe situation of the epidemic boosts the demand for these resources. In this case, the local people may have higher subjective demand for psychological intervention to address the psychological discomfort caused by the epidemic.

Therefore, in the event of a major public health emergency, medical services, good infrastructure, scientific information feedback, and high government credibility are essential foundations for easing people’s tension and decreasing people’s perception of risk.

### 5.2. Influencing Factors of the Demands for a Psychological Intervention

Psychological intervention demand is related to various factors, such as psychological state, risk perception, gender, education level, age, gender, occupation type, residence, family size, risk perception, psychological state, education level, and fixed expenditure.

The stronger the risk perception, the greater the demand for psychological intervention. This is consistent with the results of previous studies in Japan and Taiwan, China [36,37] because people with a strong risk perception are often more sensitive to changes in the external environment. Their psychological state is unstable with the changes of the environment, which leads to the need for more psychological interventions.

Compared with females, males have a greater need for psychological intervention during the epidemic. However, previous studies have shown that women suffered more mental health damage during the outbreak [38,39]. The results of this study may further promote the development of psychological intervention systems. We found that men may actually need more psychological support and psychological intervention, even though they suffer less psychological damage than women.

The educational level is closely related to the demand for psychological intervention. Previous studies have demonstrated that people with lower levels of education tend to have poorer mental health during the outbreak [40]. This further explains the current research that people with lower education levels are more in need of psychological interventions. Therefore, in the event of major public health events, the less educated population is the target that we need to pay more attention to in a psychological intervention.

People with a fixed expenditure demand less psychological intervention, which is a new finding of this study. On the one hand, this may be related to Chinese people’s traditional consumption habits and awareness of risks. Generally speaking, for the Chinese public, the demand for saving is greater than the demand for consumption [41], and people with fixed expenditure are more risk-conscious. As such, they may be prepared for any risk at any time. In addition, the epidemic happened during the Chinese Spring Festival, when people normally save more money to cover New Year’s expenses. Under the dual influence of traditional festivals and family savings, people’s preliminary preparation can meet the temporary expenditure demands, thus, reducing the psychological distress and psychological intervention demands. On the other hand, the Chinese government issued a new policy during the epidemic period, requiring financial institutions to give proper preferences on credit policies (for relevant personnel and those who have lost income sources due to the epidemic), flexibly adjust the repayment arrangements of personal credit, such as housing loans, credit cards, and reasonably postpone the repayment period [42]. These policies have greatly decreased people’s psychological distress caused by fixed expenses, thereby, reducing people’s demands for a psychological intervention.

In the elderly group, the higher the age of the elderly, the more intense their psychological intervention demands will be. This correlates to previous research that older people experienced greater psychological trauma and stress during the SARS epidemic [43]. We found that, compared with the 50–59 aged group, the 60–69 age group may demand more psychological intervention treatment due to their own physical quality, and problems such as bad moods caused by their inability to deal with the impact of the outside world.

Previous studies have presented that, under the influence of the epidemic, entrepreneurs’ optimism is significantly lower than that of non-entrepreneurs, and they also show worry and anxiety [44]. This study further found that entrepreneurs have a stronger need for psychological intervention than other professions, since entrepreneurs are more affected by various uncertainties and have greater demand for psychological intervention. Besides, the probability of psychological intervention demand of students is smaller than that of other professionals. This can explain that, after the outbreak of the epidemic, most students in China are on winter holiday at home, and the life pressure and living radius of students are relatively small, contributing to low demands for a psychological intervention.

The larger the family size, the smaller the demand for psychological intervention. The possible reason lies in that people are at home due to the lockdown policy in this epidemic, and external entertainment activities are greatly reduced. A research in Italy showed that the family is a protective factor for mental health, and those who have a large family reported higher perceived mental health [39]. Additionally, people with more family members are more likely to perform family activities at home to relieve their discomfort, reducing the demand for psychological intervention. On the contrary, residents with smaller families may be isolated at home for a long time, owing to the epidemic prevention and control requirements, resulting in a lack of communication with the outside world. Previous research in the UK has shown that people who live alone experienced more loneliness during the epidemic [45]. Those feelings, such as loneliness and negativity, may create a requirement for a psychological intervention.

Therefore, our findings suggest that, in the development of specific psychological intervention practices, more attention should be paid to special groups, such as the elderly, people with low education levels, rural residents, and residents who live alone or with smaller families. Furthermore, from the perspective of individuals’ psychological conditions, individuals’ needs, other attributes, and characteristics, differentiated psychological intervention programs should be established according to individual conditions.

### 5.3. Influencing Factors of Psychological Intervention Methods

The study revealed that risk perception, psychological status, age, gender, and family size would affect people’s choice of psychological intervention methods.

The psychological intervention method of “self-persuasion” has a higher requirement for individual psychological quality. The method of “psychological counseling under professional guidance” indicates that individuals should counsel and open their minds to psychological experts. The method of “communication and counseling among homogeneous groups” requires communication and guidance with classmates, colleagues, and other groups. People with a poor psychological status are often unwilling to choose the above three psychological intervention methods. In the case of a negative mental state, they are more reluctant to relax their expression or communicate with others [46], which results in the rejection of the above psychological intervention approaches.

People who have a higher risk perception of the epidemic tend to believe that their lives are threatened. Facing a highly contagious epidemic, they are more likely to have negative emotions, such as worry and hypochondria, which leads to the failure of “self-persuasion.” Similarly, communicating with colleagues, classmates, and other groups is the main way for “communication and counseling among homogeneous groups.” Previous studies had revealed that homogenous groups are more likely to communicate and talk, which can relieve psychological stress [47]. Nevertheless, the present study found that the stronger the people’s risk perception, the less willing they were to choose “communication and counseling among homogeneous groups.” Because the more risk people perceive, the more sensitively they will respond to external information—i.e., information about the epidemic, which may exacerbate psychological discomfort.

This is a new finding that older people will not choose “communication and counseling among homogeneous groups” during the epidemic. The public is not allowed to go out or even gather to talk to ensure the effective prevention and control of the epidemic in China. Therefore, Internet social applications such as “QQ,” “WeChat,” “Facebook,” and other Internet platforms are the primary tools used by the “communication and counseling among homogeneous groups” during this outbreak. Nevertheless, older people have a strong sense of strangeness to the Internet-based social software, resulting in certain communication barriers. A study in Switzerland showed that some older people feel excluded from society because they do not use the Internet to socialize [48]. In fact, older people who participate in online social networking with homogeneous groups can significantly reduce their feelings of loneliness [49]. Therefore, the elderly do not like the psychological intervention method of “communication and counseling among homogeneous groups” because, in the special environment, the lack of skills makes the elderly unable to communicate with the homogeneous people. The findings suggest that psychological interventions should take into account the availability of older population’s existing skills. On the other hand, it is necessary to develop a psychological intervention platform suitable for the elderly to achieve the best impact of a psychological intervention.

“Other types of counseling” mainly include certain religious beliefs and Dharma assembly to alleviate psychological pressure. A new finding in our study show that the larger the family size of a group, the lower the probability of selecting “other types of counseling (such as pujas and religious activities).” Previous studies have indicated that religious preferences are influenced by individual differences and family members [50]. The more family members there are, the more differences there will be in their choice of religion and Dharma Association. It is difficult to reach a consensus. On the other hand, the more family members there are, the more ways they can relax and relieve stress, and the less likely they are to opt for “other types of counseling (such as pujas and religious activities)”.

A new finding of the present study was that males tended to choose “other types of counseling (such as pujas and religious activities)” to meet their psychological intervention demands. Males are influenced by Chinese traditional thought: “Men focus on outdoor business, while a woman’s work centers around the indoors.” Working outside the home may enable males to obtain more information than women, which may make males prefer to enhance psychological intervention through diversified contents and forms. Males, on the other hand, may be more inclined to participate in religious activities or rituals to ease psychological discomfort, which is different from previous studies where “females are more likely to participate in religious activities” [51].

Surveys demonstrate that there are still many individuals who do not know about psychological interventions. Crisis education and psychological intervention should be strengthened in the future, and self-counseling should be performed when necessary.

There are several limitations in the research methods. (1) In this paper, the psychological symptoms of the general public in a specific period was measured under special circumstances, and the results could not reflect the changes of the general public in China under COVID-19. (2) Online questionnaire survey of snowball sampling method was adopted to collect sample data. The proportion of young people aged 20–29 in the sample is too large, making the data results have a selective bias. (3) Due to the sudden nature of the epidemic, this survey adjusted the options of the questionnaire appropriately based on the existing questionnaire and scale studies as well as the current actual situation in China to investigate the characteristics of temporary psychological changes of the public during the epidemic (Passed expert evaluation). Despite the above limitations, the author collected and analyzed the large sample data of psychological symptoms of the general public in the period of continuous change after the outbreak of the epidemic in China for one month, and the results of the research are still of significant reference value for the formulation and implementation of psychological intervention and prevention as well as control measures for the general public under COVID-19 in China.

## 6. Conclusions

It is of vital significance to identify the differences among individual demands for psychological intervention as well as a psychological intervention mechanism of mode selection. In this study, the differences in psychological intervention needs and choice of psychological intervention methods of individuals with different psychological conditions and personal characteristics during the COVID-19 epidemic were explored. The discussion part of the study can be used as research resources to provide a decision-making basis for the construction of a psychological intervention system in psychological crisis management during the post-epidemic prevention and control period, as well as scientific guidelines and practical reference for addressing psychological stress of similar sudden crisis events in the future.

Particularly, the individual characteristics and the relationship between psychological intervention research were added to this study. According to the research on psychological intervention system construction in our country, the following suggestions for the future are proposed. (1) It is necessary to build a diversified psychological intervention mechanism in the future. (2) The government should build a permanent psychological crisis emergency management mechanism and improve the government’s crisis management ability. Meanwhile, infrastructure construction and public service levels in all regions should be continuously improved. (3) The government should strengthen the publicity and knowledge popularization of methods for handling public health emergencies, and take psychological intervention as a daily education content.

## Figures and Tables

**Table 1:** Variable settings and variable assignment.

Variable Name	Variable Assignment	Med	SD	I-Q Range		
				25	50	75
Psychological intervention demand (*Y*)	1 = yes, 0 = no	0.00	0.02	0.00	0.00	0.00
Age ($x_{1}$)	1 = under 20 years old, 2 = 20–29 years old, 3 = 30–39 years old, 4 = 40–49 years old, 5 = 50–59 years old, 6 = over 60 years old	2.00	1.13	2.00	3.00	3.00
Gender ($x_{2}$)	1 = male, 2 = female	2.00	0.49	1.00	2.00	2.00
Education level ($x_{3}$)	1 = primary school and below, 2 = junior high school, 3 = Senior high school or technical secondary school, 4 = junior college and above	4.00	0.46	4.00	4.00	4.00
Residence ($x_{4}$)	1 = countryside, 2 = urban centre, 3 = urban suburb, 4 = urban centre	2.00	1.26	1.00	2.00	4.00
Family size ($x_{5}$)	number of people living in a family during the outbreak	4.00	1.36	3.00	4.00	5.00
Occupation ($x_{6}$)	1 = employees of enterprises and public institutions, 2 = middle-level and above leading cadres, 3 = entrepreneurs, 4 = students, 5 = farmers, 6 = others	4.00	1.73	1.00	4.00	4.00
Unit of nature ($x_{7}$)	1 = no fixed work unit, 2 = public sector, 3 = private sector, 4 = no job	3.00	1.05	2.00	3.00	4.00
Fixed income ($x_{8}$)	Do you have a fixed monthly income? (1 = Yes, 0 = no)	0.00	0.49	0.00	0.00	1.00
Fixed expenditure ($x_{9}$)	Whether there are fixed monthly expenses (1 = Yes, 0 = no)	1.00	0.50	0.00	1.00	1.00
Cumulative number of confirmed cases ($x_{10}$)	with February 20, 2020 as the node	-	-	-	-	-
Risk perception ($x_{11}$)	Likelihood of susceptibility to new pneumonia 1 = none, 2 = low, 3 = hard to judge, 4 = high, 5 = very high	2.00	0.74	1.00	2.00	2.00
Psychological status ($x_{12}$)	Psychological status rating (1–5 = Level 1–5 pressure)	3.00	1.42	2.00	3.00	4.00

Note: “Cumulative number of confirmed cases” is a discontinuous variable, and the situation varies from province to province.

**Table 2:** Variable settings and variable assignment.

Variable Name	Variable Assignment	Med	SD	I-Q Range		
				25	50	75
Mode of psychological intervention (*Y*)	1 = Self-persuasion, 2 = Psychological counseling under professional guidance, 3 = Communication and counseling among homogeneous groups, 4 = “Individual-social” family relationship communication facilitation, 5 = Other types of counseling, 6 = Do not understand the method of psychological counseling for the time being	2.00	1.55	1.00	2.00	3.00
Age ($x_{1}$)	1 = under 20 years old, 2 = 20–29 years old, 3 = 30–39 years old, 4 = 40–49 years old, 5 = 50–59 years old, 6 = over 60 years old	2.00	1.15	2.00	2.00	3.00
Gender ($x_{2}$)	1 = male, 2 = female	2.00	0.50	1.00	2.00	2.00
Education level ($x_{3}$)	1 = primary school and below, 2 = junior high school, 3 = senior high school or technical secondary school, 4 = junior college and above	4.00	0.69	4.00	4.00	4.00
Fixed expenditure ($x_{4}$)	Is there a fixed monthly expenditure? (1 = Yes, 0 = no)	0.00	0.49	0.00	1.00	1.00
Family size ($x_{5}$)	number of people living in a family during the outbreak	4.00	1.51	3.00	4.00	5.00
Risk perception ($x_{6}$)	Likelihood of susceptibility to new pneumonia (1 = none, 2 = low, 3 = hard to judge, 4 = high, 5 = very high)	4.00	0.96	2.00	2.00	3.00
Psychological status ($x_{7}$)	Psychological status rating 1–5 = Level 1–5 pressure	1.00	1.34	3.00	4.00	5.00

**Table 3:** Statistics of individual characteristics of respondents.

Baseline Characteristic	Frequency	Proportion (%)
**Gender**		
Male	9695	40.10
Female	14,493	59.90
**Age**		
<20	4272	17.70
20–29	11,195	46.30
30–39	286	17.40
40–49	255	12.50
50–59	203	5.30
>60	196	8.00
**Vocational type**		
Employees of enterprises and public institutions	6704	27.70
Middle management	1459	6.00
Entrepreneurs	652	2.70
Students	12,845	53.10
Farmers	453	1.90
Retired	262	1.10
Other	1813	7.50
**Education level**		
Primary school and below	148	0.60
Junior school	707	2.90
High school or technical secondary school	1514	6.30
College degree or above	21,819	90.20
**Residence**		
Rural	7726	31.90
Town center	4769	19.70
City suburb	3266	13.50
The city center	8427	34.80
**Nature of units**		
Unfixed unit	1912	7.90
The public sector	8627	35.70
The private sector	2953	12.20
No work	10,696	44.20
**Fixed income**		
Yes	10,039	41.50
No	10,149	58.50
**Fixed expenditure**		
Yes	12,363	51.10
No	11,825	48.90
**Family size**		
1–2	2652	11.00
3–4	14,517	60.00
5–7	7019	29.00

**Table 4:** The choice of public psychological intervention mode.

Psychological Counseling	Choose This Mode/%	Only Choose This Mode/%
Self-persuasion (e.g., reading, meditation)	63.10%	47.50%
Psychological counseling under professional guidance (such as individual communication with professionals)	40.35%	26.30%
Communication and counseling among homogeneous groups (e.g., colleagues, classmates, etc.)	28.50%	7.20%
“Individual-social” family relationship communication facilitation (e.g., friends, relatives, etc.)	28.30%	8.30%
Other types of counseling (such as religion and dharma association)	6.50%	2.20%
Don’t understand the way of psychological counseling for the time being	9.90%	8.50%

**Table 5:** Model estimation results of influencing factors of psychological intervention demand of the public.

Effect	β	SE	W	Exp(B)	Confidence Intervals (95%)	
					LL	UL
Age = under 20	−0.20	0.39	0.27	0.82	0.38	1.72
Age = 20–29	−0.20	0.38	0.27	0.82	0.38	1.67
Age = 30–39	−0.37	0.37	0.96	0.69	0.33	1.31
Age = 40–49	−0.57	0.37	2.36	0.56	0.26	1.13
Age = 50–59	−0.67 *	0.36	3.14	0.51	0.24	1.04
Gender = male	0.35 ***	0.07	28.42	1.42	1.26	1.63
Education level	−0.30 ***	0.07	19.51	0.74	0.65	0.85
Residence = country	0.17 *	0.10	3.36	1.18	0.99	1.41
Residence = town center	0.08	0.10	0.75	1.09	0.90	1.31
Residence = Outskirts of town	0.06	0.10	0.28	1.06	0.87	1.30
Family size	−0.04 *	0.02	3.04	0.96	0.92	1.01
Fixed income = yes	−0.07	0.13	0.26	0.93	0.72	1.22
Fixed expenditure = yes	−0.19 ***	0.07	6.81	0.83	0.71	0.94
Unit nature = no fixed unit	0.18	0.12	2.25	1.20	0.94	1.51
Unit nature = public sector	−0.10	0.12	0.62	0.91	0.71	1.14
Unit nature = private sector	−0.20	0.14	1.92	0.82	0.63	1.09
Type of occupation = employees of enterprises and institutions	−0.07	0.14	0.22	0.94	0.72	1.24
Type of occupation = middle-level cadre	0.06	0.18	0.12	1.06	0.76	1.52
Type of occupation = entrepreneur	0.32 *	0.18	3.02	1.38	0.96	1.99
Type of occupation = student	−0.28 *	0.15	3.27	0.76	0.57	1.03
Type of occupation = farmer	0.24	0.22	1.14	1.27	0.79	1.89
Psychologic status	0.49 ***	0.03	360.84	1.64	1.56	1.73
Risk perception	0.36 ***	0.04	80.97	1.44	1.33	1.56
Cumulative number of confirmed cases	0.00	0.00	0.61	1.00	1.00	1.00
Constant	−4.00	0.47	71.62	0.02	0.38	1.72

Note: ***, * indicate statistical significance at the 1%, 5%, and 10% levels, respectively.

**Table 6:** Estimation results of influencing factors of choice of psychological intervention mode.

Psychological Counseling	Effect	β	SD	W	Exp(B)	Confidence Intervals (95%)	
						LL	UL
self-persuasion	Age	−0.24	0.15	2.42	0.79	0.59	1.06
	Gender = male	0.55	0.34	2.58	1.73	0.88	3.37
	Education level	−0.01	0.23	0.00	0.99	0.63	1.55
	Fixed expenditure = no	0.19	0.36	0.28	1.21	0.60	2.42
	Family size	−0.17	0.11	2.47	0.85	0.69	1.04
	Psychological status	−0.35 **	0.14	5.83	0.71	0.53	0.94
	Risk perception	−0.45 ***	0.17	6.75	0.64	0.45	0.90
Psychological counseling under professional guidance	Age	−0.28 *	0.16	3.11	0.75	0.55	1.03
	Gender = male	0.10	0.36	0.08	1.11	0.55	2.24
	Education level	0.12	0.25	0.22	1.13	0.69	1.84
	Fixed expenditure = no	−0.02	0.37	0.00	0.98	0.47	2.04
	Family size	−0.18	0.11	2.58	0.84	0.67	1.04
	Psychological status	−0.36 **	0.15	5.73	0.70	0.52	0.94
	Risk perception	−0.17	0.18	0.93	0.84	0.59	1.20
Communication and counseling among homogeneous groups	Age	−0.62 ***	0.22	8.04	0.54	0.35	0.83
	Gender = male	0.72	0.46	2.51	2.06	0.84	5.05
	Education level	−0.15	0.31	0.24	0.86	0.47	1.58
	Fixed expenditure = no	−0.58	0.49	1.38	0.56	0.21	1.47
	Family size	−0.27 *	0.15	3.20	0.77	0.57	1.03
	Psychological status	−0.39 **	0.18	4.63	0.68	0.47	0.97
	Risk perception	−0.57 **	0.24	5.50	0.56	0.35	0.91
Personal-social family relationship communication counseling	Age	−0.31	0.20	2.42	0.74	0.50	1.08
	Gender = male	0.30	0.44	0.48	1.36	0.58	3.19
	Education level	−0.10	0.29	0.11	0.91	0.52	1.60
	Fixed expenditure = no	0.24	0.45	0.27	1.27	0.52	3.09
	Family size	−0.22	0.140	2.59	0.80	0.61	1.05
	Psychological status	−0.11	0.18	0.37	0.90	0.63	1.28
	Risk perception	0.06	0.22	0.07	1.06	0.69	1.62
Other types of counseling	Age	−0.50	0.30	2.66	0.61	0.34	1.11
	Gender = male	1.66 **	0.75	4.91	5.24	1.21	22.67
	Education level	−0.31	0.44	0.48	0.73	0.31	1.752
	Fixed expenditure = no	−0.25	0.73	0.12	0.78	0.19	3.22
	Family size	−0.52 **	0.24	4.56	0.60	0.37	0.96
	Psychological status	−0.31	0.26	1.42	0.73	0.44	1.22
	Risk perception	−0.29	0.35	0.70	0.75	0.38	1.48

Note: ***, ** and * indicate statistical significance at the 1%, 5%, and 10% levels, respectively.

## Data Availability

Data available in a publicly accessible repository. The data presented in this study are openly available in [FigShare] at [10.6084/m9.figshare.14501109].
